# The impact of the COVID-19 pandemic on women seeking fertility treatment: the patient’s perspective

**DOI:** 10.1007/s00404-021-06379-y

**Published:** 2022-01-20

**Authors:** Shona Wedner-Ross, Cordula Schippert, Frauke von Versen-Höynck

**Affiliations:** Department of Obstetrics and Gynecology, Hannover Medical School, Carl-Neuberg-Straße 1, 30625 Hannover, Lower Saxony Germany

**Keywords:** COVID-19 pandemic, Stress, Emotional impact, Fertility treatment, Pregnancy

## Abstract

**Purpose:**

This study sought the views of women with impaired fertility on the impact of the COVID-19 pandemic on their fertility treatment and psychological wellbeing.

**Methods:**

A cross-sectional, anonymous, online questionnaire was completed in June–December 2020 by 249 women attending fertility clinics across Germany. All women seeking treatment in fertility clinics were eligible to participate. The online survey covered questions about the patient’s quality of life, their opinions about the professional societies’ recommendations and their effects as well as any concerns about infection with SARS-CoV-2.

**Results:**

Three-quarters of participants disagreed with the pausing of fertility treatments. Women who participated from October to December 2020, when the incidence rate was high, were as likely to disagree as participants that participated from June to September 2020 (73% vs 79%, *p* = 0.3). Seventy-two participants (29%) had their appointments cancelled. Nearly all (97%) reported being upset by this, with 40 (56%) reporting that they were extremely or very disappointed about the cancellation. Women who had to wait 10 weeks or longer were more likely to be upset by the postponement or cancellation of their appointment than women who had to wait a shorter amount of time (*p* = 0.01). Many participants (41%) were worried about possible negative effects a SARS-CoV-2 infection might have related to their fertility, pregnancy or unborn child.

**Conclusion:**

Postponement of treatments increased distress among patients and should be avoided when possible. Fertility clinics must provide information about the current state of knowledge of SARS-CoV-2 infections in pregnancies and options for immunization.

**Supplementary Information:**

The online version contains supplementary material available at 10.1007/s00404-021-06379-y.

## Introduction

In December 2019, the first outbreak of the novel Corona Virus Disease 2019 (COVID-19) took place in Wuhan, Hubei Province, China [1]. From there, the virus quickly spread across the world and has at time of writing infected over 185 million globally, over 4 million of whom have died [2]. As a result, many countries, including Germany, implemented measures to stem the spread of disease such as advice to stay at home, travel restrictions and the closure of non-essential businesses. In spring 2020, the European Society of Human Reproduction and Embryology (ESHRE) followed by the German Society for Reproductive Medicine (DGRM) recommended that all fertility treatments should be postponed as the effects of COVID-19 on maternal and foetal health were unknown and in an attempt to lessen the burden on the health care system [3]. On account of this, most fertility treatment centres in Germany offered limited services or closed their services completely from mid-March to early-May 2020 resulting in many patients having their treatment postponed or abandoned. Although most centres have returned to treating patients under new guidelines, the COVID-19 pandemic is evolving continuously, causing uncertainty about the permanence of the reopening or the further cancellation of treatments. Additionally, the appearance of many variants of the SARS-CoV-2 virus is sure to add uncertainty as vaccine efficacy may be reduced and virus properties are not yet known [4].

Infertility treatment is time sensitive as women’s fecundity drastically reduces above the age of 32 [5], making it possible that delays in service are stressful for affected women and couples. Additionally, infertility has been shown to be a major stressor and women with infertility are more likely to suffer from anxiety and depression [6]. Thus, it is important that psychological effects of the postponement of fertility treatments are studied within this already vulnerable population. Previous studies in Canada and the United States, Italy and Israel have shown that postponing fertility clinic appointments can cause psychological distress, with most patients in those studies saying that they would have preferred to continue treatment despite the potential risk of infection from meeting other patients and clinic staff and travelling to clinic appointments [7–9]. To date, no similar studies in Germany have been reported. In addition, this study was conducted over a longer period of time than the previous studies, allowing the effect on the opinions and concerns of participants of changes in the COVID-19 incidence rate to be assessed. Furthermore, concerns that fertility clinic patients might have about the possible consequences of a SARS-CoV-2 infection on fertility and pregnancy have not been studied previously.

This cross-sectional survey was conducted throughout Germany to investigate the emotional and psychological effects of the suspension of fertility treatments and what characteristics made an individual more likely to suffer emotional distress. Our study also explored patients’ concerns regarding possible negative effects that an infection with SARS-CoV-2 might have on their health during pregnancy and the health of their unborn child.

## Method

### Participants

Women seeking fertility treatment from any fertility clinic within Germany during the period from 1st March to 19th December 2020 were eligible to take part in the survey. This period spanned from when all fertility clinics were closed (mid-March to early-May 2020) until after their reopening (early-May till the end of the study period). Participants were recruited through leaflets placed in fertility treatment centers across Germany, telephone or through in-person contact by study personnel. An advertisement for the study including the link to the survey was posted twice in an online support group on Facebook. The questionnaire itself was available online only and was accessible from 6th June to 19th December. Participants were asked to access the survey via a QR-Code on a study leaflet or via a link sent to them by e-mail.

A total of 597 participants clicked on the survey-link, 303 of whom proceeded to answer at least part of the questionnaire. Three criteria had to be met for a questionnaire to be included in the analysis. Participants had to have given their written, online consent; they had to have reached the last page of the questionnaire; and they had to have completed at least 50% of the questions. Overall, 3 of the women who started the questionnaire did not give their written consent, 3 completed less than 50% of the questions, and 255 participants reached the last page of the questionnaire, leaving a total of 249 participants (82% of the women who started the questionnaire) who contributed data to the analysis.

### Questionnaire

This cross-sectional, anonymous, online survey was created on the “SoSci Survey” platform. The survey was composed of three parts and was available in German. A translated version of the survey can be found in Supplemental Material. The first part collected information on general participant characteristics. This included demographic information such as age, education status and self-assessed quality of life and general fertility information (e.g., the length of time the woman had been trying to conceive and the type of treatment they had received) as well as problems they had faced related to the COVID-19 pandemic, such as financial problems or stress.

The second part assessed the opinions of participants on the suspension of fertility treatments that had occurred due to the COVID-19 pandemic. Participants were asked if they agreed with, disagreed with or were undecided about the decision to suspend all fertility treatments. Participants were asked if they had been affected by the suspension. If affected, participants were then asked about the emotional effect of the postponement of their treatment using a five-point rating scale (1 = extremely disappointed to 5 = not at all disappointed).

The third part of the survey assessed any worries or concerns that the woman might have about possible negative effects that an infection with SARS-CoV-2 could have on their pregnancy or their unborn child. Here the specific worries were listed and participants had to report their degree of agreement with the statement that they were worried about that issue using a five-point Likert-scale (1 = strongly agree to 5 = strongly disagree).

### Data analysis

First, the basic frequencies of every question were calculated. Categorical variables were summarised with numbers and percentages, continuous variables were summarised using means and standard deviations (SDs). Participants that met all three inclusion criteria but did not answer specific questions were excluded from the basic frequency analysis as well as from further univariate and multivariate analysis that included that particular question.

Afterwards univariate analyses, using the Pearson chi-squared test for homogeneity, were applied to test for the association between independent variables and the emotional impact of the suspension of fertility treatment reported by the woman, as well as the woman’s general degree of agreement with the decision to suspend all fertility treatments. Independent variables included participant characteristics (e.g., age, education status, self-assessed quality of life), fertility information (e.g., length of time the woman had been trying to conceive) and problems related to the COVID-19 pandemic (e.g., financial problems or stress). Binary multivariate regression models were constructed to test the independent association of all variables whose univariate association was unlikely to have been due to chance (*p* < 0.1), with (a) the emotional impact of the suspension of fertility treatments and (b) the woman’s general degree of agreement with the decision to suspend all fertility treatments because of the pandemic. Data analysis was conducted using IBM SPSS Statistics 27.

Ethics approval was obtained from the ethics committee of Hannover Medical School.

## Results

### Demographic information

A total of 303 women took part in the survey; 249 met all three of the inclusion criteria and were included in the analysis.

The characteristics of the study population are reported in Table 1. The mean age was 34 years (SD = 4.37). Participants were highly educated with 75% having completed tertiary education. Previous pregnancies were reported by 113 (45%) of the participants, however, a high proportion of these women reported that they had had no live births (52%) and the great majority of the others (43% of the total who had been pregnant) had had one live birth. A high proportion of the women who had been pregnant reported having had at least one miscarriage (56%). Most had been trying to conceive for over 2 years, with 43% having been trying to conceive between two and less than 5 years and 21% having been trying to conceive for 5 years or more. Over half (*n* = 135) had already received at least one type of fertility treatment; 58% had had at least one in-vitro fertilisation (IVF) or intracytoplasmic sperm injection (ICSI) cycle, 29% had had at least one intrauterine insemination (IUI) cycle and 21% had had at least one frozen embryo transfer cycle.

**Table 1:** Characteristics of the study population

Characteristics	*n* (%)
Age (*n*: 247)	
< 30	50 (20.2)
30–35	106 (42.9)
36–40	67 (27.1)
> 40	24 (9.7)
Highest level of education completed (*n*: 247)	
Completed tertiary education	186 (75.3)
Completed secondary education	58 (23.5)
Still in education	3 (1.2)
Previous pregnancies (*n*: 249)	
Yes	113 (45.4)
No	136 (54.6)
Number of live births reported by the 113 women who reported having had at least one previous pregnancy (*n*: 104)	
0	55 (51.9)
1	45 (42.5)
2	3 (2.8)
3	1 (0.9)
Number of miscarriages (*n*: 106)	
0	43 (40.6)
1	44 (41.5)
2	7 (6.6)
3	10 (9.4)
4	2 (1.9)
Number of abortions (*n*: 106)	
0	89 (84.0)
1	14 (13.2)
2	2 (1.9)
3	1 (0.9)
Number of ectopic pregnancies (*n*: 106)	
0	94 (88.7)
1	9 (8.0)
2	3 (2.8)
Length of time trying to conceive (*n*: 234)	
< 1 year	17 (7.3)
1 year till less than 2 years	67 (28.6)
2 till less than 5 years	100 (42.7)
> 5 years	50 (21.4)
Previously received treatments (*n*: 135)	
Timed intercourse	56 (41.5)
Intrauterine insemination	39 (28.9)
In-vitro fertilisation/intracytoplasmic sperm injection	78 (57.8)
Frozen embryo transfer	28 (20.7)
Other	10 (7.4)
Type of appointment postponed or cancelled (*n*: 249)	
First appointment	135 (54.2)
Follow-up appointment	114 (45.8)
Self-assessed quality of life (*n*: 248)	
Very good	54 (21.8)
Good	136 (54.8)
Neither good nor bad	47 (19.0)
Bad	10 (4.0)
Very bad	1 (0.4)
Stress due to the COVID-19 pandemic (*n*: 235)	
Strongly agree and agree	95 (40.4)
Neither agree nor disagree	97 (41.3)
Tend to disagree or disagree	43 (18.3)
Financial problems due to the COVID-19 pandemic: (*n*: 249)	
Yes	50 (20.1)
No	199 (79.9)

### Emotional impact of the COVID-19 pandemic and the suspension of fertility treatment

While three quarters of the participants rated their quality of life as very good or good, 40% strongly agreed or agreed to be stressed due to the COVID-19 pandemic. One-fifth reported that they had had financial problems due to the COVID-19 pandemic.

**Fig. 1:**
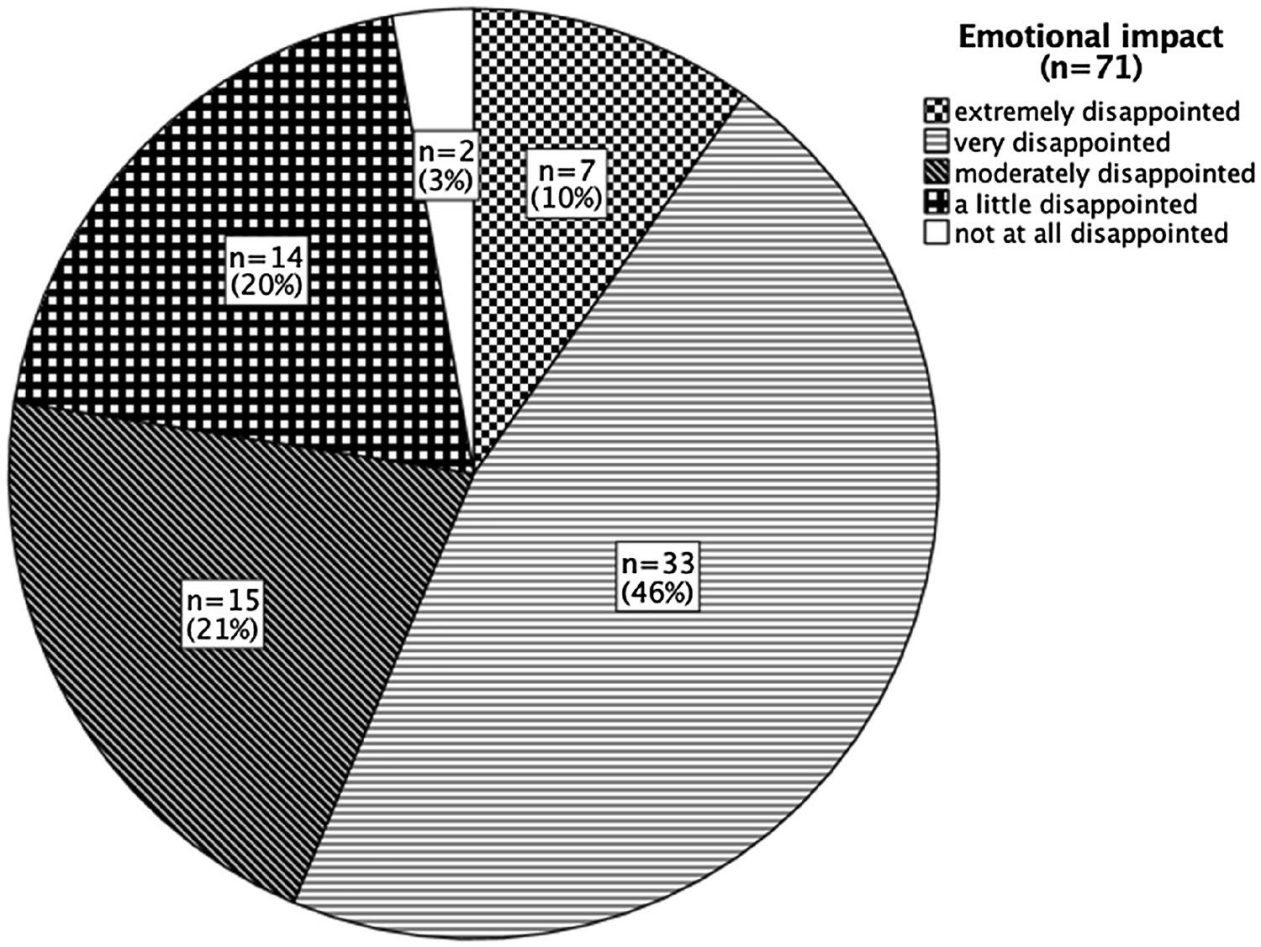
Emotional impact due to the postponement of an appointment/treatment due to the COVID-19 pandemic. Extremely disappointed being the equivalent to the loss of a child

More than a quarter (29%) of the participants reported that their fertility treatment had been cancelled due to the COVID-19-related suspension of fertility treatments. Table 2 shows the type of treatments that were cancelled. Nearly all participants (97%) said they were disappointed about the postponement of their treatment, with 10% reporting being extremely disappointed (extremely disappointed being the equivalent to the loss of a child) and 46% reporting being very disappointed. Figure 1 depicts the emotional impact due to the postponement of an appointment/treatment.

**Table 2:** Type of treatments cancelled or postponed due to the COVID-19 pandemic

	*n* (%)
Cancelled or postponed treatment (*n*: 242)	
Yes	71 (28.5)
Type of treatment:	
Timed intercourse	8 (11.3)
Intrauterine insemination	8 (11.3)
In-vitro fertilisation/intracytoplasmic sperm injection cycle	28 (39.4)
Frozen embryo transfer cycle	3 (4.2)
Operation (e.g., hysteroscopy, laparoscopy)	4 (5.6)
First consultation	7 (9.9)
Treatment cancelled due to other reasons	5 (7.0)
Unknown	8 (11.3)

The results of the univariate analyses of the associations between participant characteristics and the emotional impact of the suspension of fertility treatment showed that participants that had their appointment postponed for longer than 10 weeks were more likely to be extremely or very disappointed than participants that had to wait a shorter amount of time (*p* = 0.01). This correlation remained independently significant after adjusting for whether the appointment was a first or a follow-up appointment (Table 3).

**Table 3:** Characteristics of participants that were either extremely or very disappointed by the postponement of their treatment by length of the postponement and appointment type and binary multivariate logistic regression analysis of the influencing factors on the emotional impact of the postponement of treatment

Variables	*n*	%	Emotional impact—univariate analysis					Binary multivariate logistic regression		
			Extremely or very disappointed		Not extremely or very disappointed		*p* (homogeneity)	Emotional impact—extremely or very disappointed		
			*n*	%	*n*	%		aOR	95% CI	*p*
Length of postponement of appointment							0.01			
2–< 5 weeks	13	18.6	6	46.2	7	53.8		0.25	0.06–1.04	0.06
5–< 10 weeks	29	41.4	12	41.4	17	58.6		0.191		0.01
10 weeks or longer	28	40.0	22	78.6	6	21.4			0.06–0.61	
Appointment type							0.02			
First appointment	7	9.9	2	28.6	5	71.4		0.321	0.05–2.13	0.24
Follow-up appointment	64	90.1	38	59.4	26	40.6				

*aOR* adjusted odds ratio, *95% CI* 95% confidence interval

While univariate analysis showed that participants whose postponed appointment would have been their first appointment were less likely to be extremely or very disappointed than those whose postponed appointment was for a follow-up appointment (*p* = 0.02) (Table 3), this association was no longer statistically significant after adjusting for the length of time of the postponement (OR = 0.32, 95% CI 0.05–2.13, *p* = 0.24).

### Participants’ opinion on the suspension of fertility treatments

Three-quarters of participants disagreed with the suspension of fertility treatments. The majority (61%) believed that the suspension of fertility treatments would negatively impact their chances of getting pregnant. The current incidence rate of COVID-19 in Germany did not appear to make a difference to the disapproval rate, as women who participated in the survey between June and September 2020 when the national COVID-19 incidence per 100,000 population ranged between 2.8 and 16.0, [10] were as likely to disagree as participants who participated between October and December 2020, when the incidence rate per 100,000 population was much higher ranging between 17.0 and 217.6 [10] (73% vs 79%, *p* = 0.3).

In the univariate analysis, four factors had an association (*p* < 0.10) with the respondents’ opinions about the decision to postpone all fertility treatments because of the COVID-19 pandemic (Table 4). Participants who disagreed with the suspension of fertility treatments had similar demographic characteristics to the participants that agreed or were undecided, except for their reported level of stress related to the COVID-19 pandemic (*p* = 0.01), self-assessed quality of life (*p* = 0.03) or whether or not their appointment had been cancelled or postponed (*p* = 0.01). The length of time they had been trying to conceive was slightly but not significantly longer (*p* = 0.06).

**Table 4:** Patients’ opinions about the decision to postpone all fertility treatments by their reported stress due to the pandemic, self-assessed quality of life, and being affected by the postponements

Variables	*n*	%	Opinion about the decision to postpone all fertility treatments				
			Agree or undecided		Disagree		*p*
			*n*	%	*n*	%	
The COVID-19 pandemic is stressful to me							0.01
Strongly agree or agree	95	40.4	32	33.7	63	66.3	
Neither agree nor disagree	97	41.3	23	23.7	74	76.3	
Tend to disagree or disagree	43	18.3	4	9.3	39	90.7	
I would describe my quality of life as good							0.03
Strongly agree or agree	178	76.1	40	22.5	138	77.5	
Neither agree nor disagree	46	19.7	12	26.1	34	73.9	
Tend to disagree or disagree	10	4.3	6	60	4	40.0	
Appointment cancelled or postponed							0.01
Yes	66	28.8	24	36.4	42	63.6	
No	163	71.2	33	20.2	130	79.8	
Length of time trying to conceive							0.06
< 1 year	17	7.3	4	23.5	123	76.5	
1–less than 2 years	67	28.6	14	20.9	53	79.1	
2–less than 5 years	100	42.7	21	21.0	79	79.0	
> 5 years	50	21.4	20	40.0	30	60.0	

Only two of these factors remained independently associated with disagreement with the decision that all fertility treatments should be paused because of the COVID-19 pandemic (Table 5). The participants that tended to disagree or disagreed with the statement “the Covid-19 pandemic is stressful to me”, were more likely to disagree with the suspension of treatments (aOR = 3.48, 95% CI 1.11–11.0, *p* = 0.03) in the adjusted analysis. Interestingly, participants whose appointment was not postponed were also more likely to disagree with the suspension of fertility treatments (aOR = 2.08, 95% CI 1.06–4.10, *p* = 0.03). In the adjusted analysis, women who said that they would “tend to disagree” or “disagree” with the statement “I would describe my quality of life as good” were borderline significantly less likely to disagree with the official decision to pause all fertility treatments because of the COVID-19 pandemic (aOR = 0.22, 95% CI 0.05,0.99, *p* = 0.05).

**Table 5:** Binary multivariate logistic regression analysis of the influencing factors on the disagreement with the decision to cancel all treatments

Variables	Disagreement with the decision to pause all fertility treatments		
	aOR	95% CI	*p*
The COVID-19 pandemic is stressful to me			
Strongly agree or agree	1		
Neither agree nor disagree	1.41	0.71–2.78	0.32
Tend to disagree or disagree	3.48	1.11–11.0	0.03
I would describe my quality of life as good			
Strongly agree or agree	1		
Neither agree nor disagree	0.98	0.44–2.19	0.98
Tend to disagree or disagree	0.22	0.05–0.99	0.05
Appointment cancelled or postponed			
Yes	1		
No	2.08	1.06–4.10	0.03
Length of time trying to conceive			
< 1 year	2.06	0.51–8.34	0.32
1–less than 2 years	2.01	0.82–5.00	0.13
2–less than 5 years	2.08	0.93–4.66	0.07
> 5 years	1		

*aOR* adjusted odds ratio, *95% CI* 95% confidence interval

### Concerns and worries about a possible infection with SARS-CoV-2

**Fig. 2:**
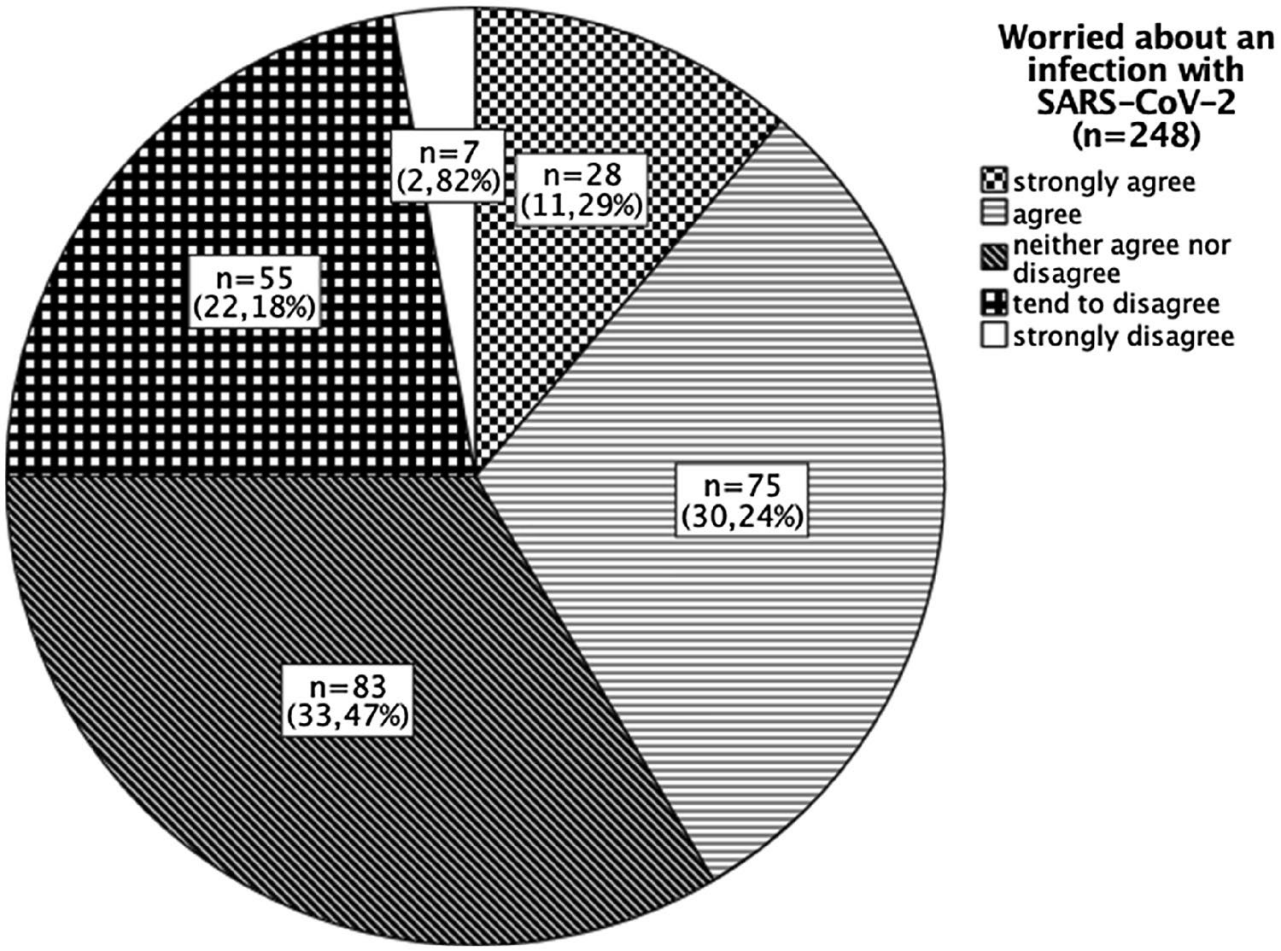
Concern regarding the impact an infection with SARS-CoV-2 would have on participant’s fertility, pregnancy or unborn child

The majority of respondents were worried about possible negative effects that a SARS-CoV-2 infection might have on their fertility, pregnancy or unborn child. As shown in Fig. 2, 11% strongly agreed and a further 30% agreed that they were concerned regarding the impact an infection with SARS-CoV-2 might have on their fertility, pregnancy or unborn child.

**Fig. 3:**
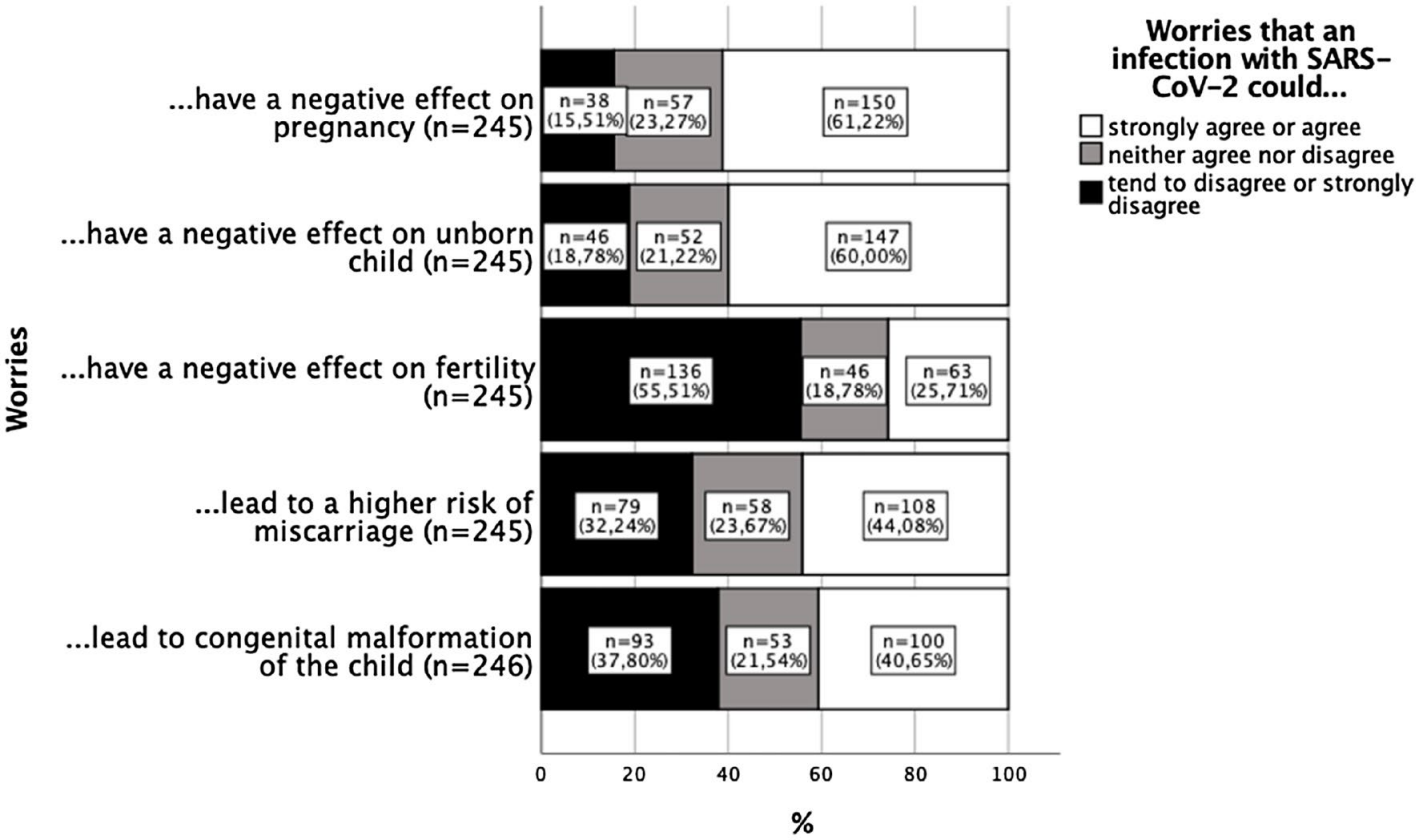
Participant’s detailed concerns about the consequences of a possible infection with SARS-CoV-2

Sixty-one percent stated they were very or moderately concerned about the negative influence of a SARS-CoV-2 infection might have on the woman’s own health during a pregnancy and 60% were very to moderately concerned about potential negative effects for the unborn child (Fig. 3). However, only 26% reported they were very or moderately concerned about the potential negative effects of an infection on fertility.

## Discussion

This cross-sectional survey sought the views of women seeking fertility treatment in Germany on the impact of the COVID-19 pandemic on three primary objectives. First, the emotional impact of the suspension of treatments due to the COVID-19 pandemic. Second, the opinion of women on the suspension of fertility treatments and lastly, worries and concerns about a possible infection with SARS-CoV-2.

While there have been studies concerning the emotional impact of the suspension of treatments [7–9, 11–13], this is the first to have been conducted in Germany where, in 2020, incidence and case fatality rates were relatively low compared to many other European countries [14]. To our knowledge, it is the first study to assess the possible worries that fertility treatment patients might have concerning an infection with SARS-CoV-2. This study is also unique in that it was conducted over a 6-month period from June to December 2020. Because of this we were able to assess whether varying COVID-19 incidence rates made a difference to the level of acceptance of the suspension of fertility treatment.

The COVID-19 pandemic has had drastic implications for the lives of people worldwide. The measures undertaken to slow the spread of disease meant that many aspects of daily life such socializing with friends, working with colleagues in an office and many leisure activities were halted suddenly and job losses or reduced income became a frequent occurrence [15]. This has been shown to increase rates of generalized anxiety, depression and distress in the general population [16]. Furthermore, infertility itself is a major cause of emotional distress [6]. It is, therefore, unsurprising that our study found that the suspension of fertility treatments has caused further stress in this already vulnerable population, with 10% reporting that the disappointment related to the suspension of their fertility treatment was “equivalent to the loss of a child”. Other studies in Israel [7], Italy [8], Northeast America and Canada [9, 11] and UK [12, 13] have also reported that the suspension of fertility treatment has caused substantial distress.

Unexpectedly, no demographic factors such as age or education status were significantly associated with the level of distress felt. However, this is in line with the findings of other studies [7, 11] and suggests that the suspension of fertility treatment causes significant distress in patients regardless of their demographic characteristics. Surprisingly, the length of time that the couple had been trying to conceive was also not significantly associated with the degree of distress reported by participants related to the cancellation of their treatment. This contradicts previous research in Canada and the United States that found a significant association between a greater length of time the woman had been trying to conceive and increased disappointment about the suspensions [11]. However, in our study, the length of postponement was associated with the level of disappointment patients felt, with patients that had to wait 10 weeks or longer were more likely to be extremely or very disappointed than patients that had to wait for a shorter amount of time.

Three-quarters (74.9%) disagreed with the suspension of fertility treatments. This figure is considerably higher than in other studies, such as studies in Israel by Ben-Kimhy et al. [7] and in the USA by Lawson et al. [17], where about half the participants disagreed with the suspension of treatments. One possible factor that could influence opinions on the suspension of treatments, is age. One could hypothesize that patients that are older might be more likely to want treatment faster and, therefore, be more likely to disagree with the suspension of treatments due to the progressive decrease in fecundity [5]. However, the mean age of 34 years in our study was younger than that in the other two studies (Ben-Kimhy: mean age = 37 years and Lawson and colleagues: mean age = 35.61 years) [7, 17]. There is also the possibility of differential selection bias between the three studies. One might hypothesize that women with stronger opinions may well have been more likely to take part. However, all three studies used similar forms of recruitment making this explanation unlikely. In our German cohort, participants were recruited either by responding to advertisements placed in social media groups on Facebook, through flyers left in fertility clinic waiting rooms, by the researchers contacting them by telephone, or through in-person recruitment by fertility clinic staff. The other two studies, in Israel and the USA, recruited by sending a request for study participation to all women in selected fertility clinics by e-mail [7, 17]. However, it is not obvious that our strategy would have led to greater (or lesser) selection bias. Cultural differences could also be a reason for the high rate of disagreement with the suspension of treatments in our study in Germany. Another potential reason might have been a difference in incidence rate, however, all three countries had similar incidence and death rates at the time of suspension of fertility treatments making this explanation also unlikely [10].

Incidence rates in our study seem to have made no significant difference to the disapproval rate. Against our expectations, women who participated in the survey between June and September 2020 when the national COVID-19 incidence was relatively low were as likely to disagree as participants who participated between October and December 2020, when the incidence rate was much higher (73% vs 79%, *p* = 0.3). Research suggests that the longer physical distancing, hygiene and masking measures persist the more likely people are to disregard the measures set by the government. This phenomenon has been called “quarantine fatigue” [18]. We suggest that any tendency to greater agreement in the later (Oct–Dec) period related to higher COVID-19 incidence might have been offset by “quarantine fatigue”. This reasoning is speculative and further research would have to be conducted to prove this hypothesis.

As expected, the amount of stress that a participant reported feeling due to the COVID-19 pandemic was associated with the disapproval rates of the suspension of fertility treatments. The participants who “tended to disagree” or “disagreed” with the statement “the COVID-19 pandemic is stressful to me” and were thus less stressed about the COVID-19 pandemic, were much more likely to disagree with the suspension of treatments (OR = 3.48, 95% CI 1.11–11.0, *p* = 0.03). People who feel less stressed about COVID-19 have been shown to be less likely to agree with and adhere to quarantine measures [19]. Thus, it is not surprising that the participants who were less stressed about the COVID-19 pandemic were more likely to disagree with measures taken to stem the spread of the SARS-CoV-2 virus such as the suspension of fertility treatments.

Interestingly, participants who had not had their treatment cancelled were more likely to disagree with the suspension of treatments in general than patients who had had their fertility treatment cancelled. We speculate that clinic staff may have taken time to inform the patients whose appointments had to be cancelled on the reasons why their treatment had been cancelled, which led to a higher agreement rate than among patients who had not had their treatment cancelled and thus did not get this explanation. This speculation is supported by the fact that Lawson and colleagues found that patients who had received written information, about the reasons why all fertility treatment was suspended showed a higher rate of acceptance of the suspension than patients who had not received any information [17].

In addition to stress regarding infertility and stress due to the COVID-19 pandemic, patients were also worried about possible negative effects that a SARS-CoV-2 infection might have on their health or the health or development of their fetus if they became pregnant. Related to their own health, women were especially worried about potential negative effects on their health during pregnancy, with 61% stating they were very or moderately concerned. As pregnancy has been shown to be an independent risk factor for severe COVID-19 [20], patients should be made aware of these risks before entering their next treatment cycle and women should be particularly encouraged to adhere to public health advice related to decreasing the risks of SARS-CoV-2 infection (physical distancing, wearing of a mask, good hygiene practices and acceptance of vaccination if offered). On the other hand, efforts should also be made to prevent inappropriate levels of stress. The majority (60%) of women were also very or moderately concerned about the potential negative effects that an infection with SARS-CoV-2 could have on their unborn child if they became pregnant. Intrauterine transmission of SARS-CoV-2 has occurred and maternal infections with severe SARS-CoV-2 have a higher risk of preterm delivery [20]. Additionally, preeclampsia and thrombo-embolic events seem to be increased among pregnant women with a SARS-CoV-2 infection [21]. Women who are trying to become pregnant should be informed of these risks and be particularly encouraged to follow public health recommendations to reduce their risk of SARS-CoV-2 infection. It must, however, be noted, that although so many participants were worried about the health of mother and child, the majority of participants still disapproved of the suspension of fertility treatments.

A quarter of women reported being very or moderately concerned about a potential negative effect of SARS-CoV-2 infection on fertility. It has been hypothesized that SARS-CoV-2 could have a negative effect on female fertility [22]. Furthermore, an infection with SARS-CoV-2 has been proven to have a negative impact on male fertility [23, 24]. Physicians should actively ask patients about concerns and worries regarding an infection with SARS-CoV-2, especially regarding their own health during pregnancy and the health of their unborn child and offer support regarding stress and anxiety due to these concerns. It would certainly also be advisable to inform patients about the current state of knowledge and ensure that physicians stay abreast of new evidence in this field.

The main limitation of this study is that we do not know the representativeness of the sample of women who participated and so cannot exclude the possibility of selection bias. However, participants were recruited from fertility treatment clinics throughout Germany and also through social media, meaning that participants are likely to have come from widespread areas in Germany. Furthermore, the demographic information of our sample is similar to that of the German population seeking fertility treatment [25]. Although the overall response rate (participants that clicked on the link and then completed the questionnaire) of 42% is relatively low, it is similar to other studies conducted on the same subject [8, 9, 17]. Additionally, our response rate is likely to be an underestimate, as people were able to click on the link multiple times or could have clicked on it and decided that they were not eligible. Also, clinic staff and study personnel clicked on the link. Additionally, the attrition rate (i.e., people who started the survey but did not finish it) was only 18%. Well-educated women are overrepresented in this study with 75% having completed tertiary education. This is, however, only slightly higher than the national average, where 65% of Germans have completed tertiary education [26]. A further limitation is that men were excluded from the study; further studies will be needed to ascertain the emotional impact of postponing fertility treatment and concerns regarding an infection with SARS-CoV-2 on men seeking fertility treatment. Furthermore, the study was cross-sectional so there is no information about patients’ views and concerns at other time points. As the COVID-19 pandemic is continuously evolving, participants’ opinions may vary over time. However, this limitation is minimised as this study was conducted over 6 months, which made it possible to capture the opinions of patients throughout that period.

In conclusion, postponement of fertility treatments increased distress among patients and should be avoided whenever possible. If unavoidable, the length of postponement should be minimised as patients that had their appointment postponed for longer than 10 weeks were more likely to experience a negative emotional impact. Patient’s were also worried about possible negative effects of an infection with SARS-CoV-2 on their health and on the health and development of their fetus if they were to become pregnant. Fertility clinics must provide information about the current state of knowledge of the potential effects of SARS-CoV-2 infections in pregnancies as well as offering support regarding anxiety and stress due to the COVID-19 pandemic.

## Supplementary Information

Below is the link to the electronic supplementary material.

Supplementary file (PDF 126 KB)

## Data Availability

Data will be made available by the corresponding author after reasonable request.
