# Supplementary material for: The impact of the COVID-19 pandemic on women seeking fertility treatment: the patient’s perspective
Source: Arch Gynecol Obstet. 2022 Jan 20;305(6):1615–24. doi: 10.1007/s00404-021-06379-y (PMC8773402; doi:10.1007/s00404-021-06379-y)
Supplement: Supplementary file 1 — Supplementary file (PDF 126 KB) [file 404_2021_6379_MOESM1_ESM.pdf]

The impact of the COVID-19 pandemic on women seeking fertility treatment: the patient´s perspective

Shona Wedner-Ross[1]; Cordula Schippert[1], Frauke von Versen-Höynck, M.D., M.S.[1*]

[1]Hannover Medical School Department of Obstetrics and Gynecology, Hannover, Lower Saxony, Germany

[*]Corresponding Author:

Prof. Dr. med. Frauke von Versen-Höynck, MD, MSc
Department of Obstetrics and Gynecology
Hannover Medical School
Carl-Neuberg-Straße 1
30625 Hannover
Germany
E-Mail: vonversen-hoeynck.frauke@mh-hannover.de
Telephone: +49 511 532 6074
Fax: +49 511 532 6081
ORCID-ID: 0000-0002-1924-5695

Supplemental Material

1. Paper version of the survey, translated into English. The survey was available to participants online on the SoSci Survey platform.

A002 – Age:

A003 – What is your highest level of education?
- Finished school without qualification
- Still in school/apprenticeship/university
- Hauptschulabschluss (lower-level secondary education)
- Realschulabschluss (mid-level secondary education)
- Fachabitur (higher-level secondary education)
- Abitur (higher-level secondary education)
- Completed apprenticeship
- University qualification (e.g., Bachelor, Masters, PHD)

A005 – How would you describe your physical health?
- very good
- good
- neither good nor bad
- bad
- very bad

A006 – How would you describe your quality of life?
- very good
- good
- neither good nor bad
- bad
- very bad

A007 – To what extent are you able to manage daily activities?
- very well
- well
- neither well nor badly
- badly
- very badly

A008 – I find the Covid-19 pandemic stressful
- strongly agree
- agree
- neither agree nor disagree
- tend to disagree
- disagree

A009 – My financial situation has got worse due to the Corona pandemic
- Yes
- No

B009 – I have been pregnant in the past
- Yes
- No (skip to B004)

B010- Number of previous pregnancies:

B003 –Number of..:

[01] Live births:
[02] Miscarriages:
[03] Abortions:
[04] Ectopic pregnancies:

B004 – I have been trying to conceive for:

- Less than one year
- 1 year till less than 2 years
- 2 years till less than 5 years
- 5 years or longer

B006 – I had already received at least one type of treatment before the Corona pandemic began.

- Yes
- No (skip to B011)

B007 – The treatment I received was:

- *Timed intercourse*
- *Intrauterine insemination*
- *In-vitro-fertilization (IVF) with or without intracytoplasmic sperm injection (ICSI)*
- *Frozen embryo transfer*
- *Other:* ________________________________

B011 – My current wish to conceive has:

- decreased
- stayed the same
- increased

B008 – My current/upcoming appointment in a treatment center is

- my first appointment
- a follow up appointment (skip to WV01)

First Appointment

EV01 – My first appointment...
- took place at the expected time
- was cancelled or postponed due to the corona virus (skip EV03)

EV02 – My first appointment
- *was conducted in the fertility treatment center (skip to EV07)*
- *was conducted by telephone (skip to EV07)*
- *was conducted by video-call (skip to EV07)*

EV03 – My first appointment was delayed by
- *1 week till less than 2 weeks*
- *2 weeks till less than 5 weeks*
- *5 weeks till less than 10 weeks*
- *10 weeks or longer*

EV04 – My postponed first appointment
- *was conducted in the fertility treatment center*
- *was conducted by telephone*
- *was conducted by video-call*

EV05 – As my appointment was postponed, I contacted other treatment centers about possible appointments
- *Yes, but they had no available appointments*
- *Yes, and I am beginning/continuing my treatment in the new treatment center*
- *No, I am glad that my chosen treatment center adhered to the guidelines.*

EV08 – How disappointed were you about the postponement of your treatment.
- *extremely disappointed (equivalent to the loss of a child)*
- *very disappointed*
- *moderately disappointed*
- *a little disappointed*
- *not at all disappointed*

EV07 – What is your opinion about the guidelines of international and national professional societies about the pausing of following treatments:

	I agree	I am undecided	I disagree
All fertility treatments	☐	☐	☐
Timed intercourse	☐	☐	☐
Intrauterine insemination	☐	☐	☐
In-vitro-fertilization (IVF) with or without	☐	☐	☐

intracytoplasmic sperm injection (ICSI)			
Frozen embryo-transfer	☐	☐	☐
Operations, such as hysteroscopy or laparoscopy	☐	☐	☐
Diagnostic tests (such as hormone analysis)	☐	☐	☐

EV09 – Before I start my fertility treatment, I would like to talk to my doctor about possible effects an infection with the SARS-CoV-2 Virus would have on my fertility treatment.

- strongly agree
- agree
- neither agree nor disagree
- tend to disagree
- disagree

EV10 – Before I start my fertility treatment, I would like to talk to my doctor about possible effects an infection with the SARS-CoV-2 Virus would have on a pregnancy.

- strongly agree
- agree
- neither agree nor disagree
- tend to disagree
- disagree

EV11 – I am worried that a delay in treatment due to the corona pandemic will worsen my chances of getting pregnant.

- strongly agree
- agree
- neither agree nor disagree
- tend to disagree
- disagree

EV12 – I am worried that I won’t be able to continue my fertility treatment on account of financial difficulties due to the corona pandemic.

- strongly agree
- agree
- neither agree nor disagree
- tend to disagree
- disagree

EV13 – I am worried, that because of the treatment delays due to the corona pandemic, I will reach the age limit for contribution towards the cost of my treatment (with state health insurance; 40 years)

- strongly agree

- agree
- neither agree nor disagree
- tend to disagree
- disagree

EV14 – I am worried that an infection with SARS-CoV-2 will…

	Strongly agree	Agree	Neither agree nor disagree	Tend to disagree	Strongly disagree
have a negative effect on fertility	☐	☐	☐	☐	☐
lead to a higher risk of miscarriage	☐	☐	☐	☐	☐
have a negative effect on pregnancy	☐	☐	☐	☐	☐
have a negative effect on the unborn child	☐	☐	☐	☐	☐
Lead to congenital malformation of the child	☐	☐	☐	☐	☐

Continue with C001

Follow-up appointment

WV01 – The reason for my current follow-up appointment is
- To plan further treatment
- To continue my current treatment
- Other reason for the consultation: ___________

WV02 – During the corona pandemic my treatment was:
- Continued as planned in the same treatment center (skip to WV06)
- Continued as planned due to transferring treatment centers (skip to WV06)
- Cancelled
- Postponed

WV03 – My treatment was postponed for
- *1 week till less than 2 weeks*
- *2 weeks till less than 5 weeks*
- *5 weeks till less than 10 weeks*
- *10 weeks or longer*

WV04 – Following treatments were cancelled or postponed due to the corona pandemic
- *Timed intercourse*
- *Intrauterine insemination*
- *In-vitro-fertilization (IVF) with or without intracytoplasmic sperm injection (ICSI)*
- *Frozen embryo transfer*
- *Other treatment:*
- *My treatment was postponed or cancelled due to other reasons.*

WV05 – How disappointed were you about the cancellation/postponement of your appointment:
- *extremely disappointed (equivalent to the loss of a child)*
- *very disappointed*
- *moderately disappointed*
- *a little disappointed*
- *not at all disappointed*

WV07 – If you had had the option, would you have started a new treatment cycle?
- *Yes, I would have started a new treatment cycle*
- *No, I would not have started a new treatment cycle*
- *I would not have been sure about whether to start a new treatment cycle or not.*

WV06 - What is your opinion about the guidelines of international and national professional societies about the pausing of following treatments:

	I agree	I am undecided	I disagree
All fertility treatments	☐	☐	☐
Timed intercourse	☐	☐	☐
Intrauterine insemination	☐	☐	☐
In-vitro-fertilization (IVF) with or without intracytoplasmic sperm injection (ICSI)	☐	☐	☐
Frozen embryo-transfer	☐	☐	☐
Operations, such as hysteroscopy or laparoscopy	☐	☐	☐
Diagnostic tests (such as hormone analysis)	☐	☐	☐

WV08 – Before starting my current treatment, I consulted a doctor about possible effects an infection with SARS-CoV-2 could have on my fertility treatment.

- ○ yes
- ○ no

WV09 - Before starting my current treatment, I consulted a doctor about possible effects an infection with SARS-CoV-2 could have on a pregnancy.

- ○ Yes
- ○ No

WV10 - I am worried that a delay in treatment due to the corona pandemic will worsen my chances of getting pregnant.

- ○ strongly agree
- ○ agree
- ○ neither agree nor disagree
- ○ tend to disagree
- ○ disagree

WV11 - I am worried that I won’t be able to continue my fertility treatment on account of financial difficulties due to the corona pandemic.

- ○ strongly agree
- ○ agree
- ○ neither agree nor disagree
- ○ tend to disagree
- ○ disagree

WV12 - I am worried, that because of the treatment delays due to the corona pandemic, I will reach the age limit for contribution towards the cost of my treatment (with state health insurance; 40 years)

- strongly agree
- agree
- neither agree nor disagree
- tend to disagree
- disagree

WV13 – I am worried that an infection with SARS-CoV-2 will…

	Strongly agree	Agree	Neither agree nor disagree	Tend to disagree	Strongly disagree
have a negative effect on fertility	?	?	?	?	?
lead to a higher risk of miscarriage	?	?	?	?	?
have a negative effect on pregnancy	?	?	?	?	?
have a negative effect on the unborn child	?	?	?	?	?
Lead to congenital malformation of the child	?	?	?	?	?

<u>Conclusion</u>

C001 – How did you find out about this study?

- In-person contact by study personnel
- Flyer in your treatment center
- Telephone contact by study personnel
- E-Mail contact by study personnel
- Advert on Facebook
- Advert of Instagram
- Support groups
- Through another patient
- Other: ___________________

C002 – Anything you would like to add?:
